# The relationship between perioperative serum albumin and contrast-induced acute kidney injury in patients after percutaneous coronary intervention

**DOI:** 10.1186/s12882-024-03608-9

**Published:** 2024-05-21

**Authors:** Dong Wang, Gaoliang Yan, Yong Qiao, Renhua Sun

**Affiliations:** 1 Department of Cardiology, School of Medicine, Southeast University, Zhongda Hospital, Nanjing, P.R. China; 2 Department of Cardiology, The First people’s Hospital of Yancheng, The Yancheng Clinical College of Xuzhou Medical University, Yancheng, P.R. China; 3 Jiangsu Key Laboratory for Design and Manufacture of Micro-Nano Biomedical Instruments, Southeast University, Nanjing, P.R. China; 4 School of Medicine, Southeast University, Nanjing, P.R. China

**Keywords:** Contrast-induced acute kidney injury, Perioperative serum albumin (&Alb), Percutaneous coronary intervention

## Abstract

**Objective:**

Contrast-induced acute kidney injury (CI-AKI) is a common complication in patients undergoing percutaneous coronary intervention (PCI). Studies have shown that perioperative serum albumin levels may play a role in the occurrence of CI-AKI. In this study, we aimed to investigate the effect of perioperative serum albumin (delta albumin or &Alb) levels on the occurrence and long-term prognosis of CI-AKI patients after PCI.

**Methods:**

A total of 959 patients who underwent PCI between January 2017 and January 2019 were selected for this study. A receiver operating characteristic curve was used to determine the optimal cut-off value of the &Alb level for predicting CI-AKI after PCI. Patients were divided into two groups based on the optimal cut-off value: the high &Alb group (&Alb ≥ 4.55 g/L) and the control group (&Alb < 4.55 g/L). The incidences of CI-AKI and major adverse cardiac events (MACEs, including all-cause death, nonfatal myocardial infarction, and target vessel revascularization) were compared between the groups. Cox regression analysis was used to identify predictors of long-term prognosis after PCI.

**Results:**

Of the 959 patients, 147 (15.3%) developed CI-AKI after PCI. The CI-AKI group had a greater level of &Alb than did the non-CI-AKI group [(6.14 (3.90–9.10) versus 3.48 (4.31–6.57), *P* < 0.01)]. The incidence of CI-AKI in the high &Alb group was significantly greater than that in the low group (23.6% versus 8.3%, *P* < 0.01). After a 1-year follow-up, the incidence of MACEs was significantly greater in the high &Alb group than in the low group (18.6% versus 14.5%, *P* = 0.030). Cox regression analysis confirmed that CI-AKI was an independent predictor of MACEs at the 1-year follow-up (HR 1.43, 95% CI 1.04–1.96, *P* = 0.028). In addition, patients with low preoperative serum albumin levels had s significantly greater incidence of MACEs than did those with high preoperative serum albumin levels (23.2% versus 19.5%, *P* = 0.013).

**Conclusion:**

In summary, high baseline &Alb levels are an independent risk factor for CI-AKI in patients after PCI. The occurrence of CI-AKI in the perioperative period is also an independent predictor of long-term prognosis after PCI. These findings highlight the importance of monitoring &Alb levels and taking steps to prevent CI-AKI in patients undergoing PCI.

## Introduction

Percutaneous coronary intervention (PCI) is a common treatment for patients with coronary artery disease (CAD). Contrast-induced acute kidney injury (CI-AKI) is defined as an acute decline in renal function following the administration of iodinated contrast media [1]. In patients undergoing PCI, CI-AKI has become a complication that is closely linked to adverse clinical events. Contrast-induced nephropathy (CIN) is associated with increased rates of morbidity, mortality, and health care costs [2, 3]. CI-AKI is defined as a 25% increase in serum creatinine levels or an absolute increase of more than 44.2 µmol/L following the administration of contrast media [4, 5]. The pathogenesis of CI-AKI is complex, and the specific pathophysiologic basis remains unclear. Increased blood viscosity, reperfusion injury, direct toxicity to renal tubule cells, and long-term vasoconstriction are closely related to the occurrence of CI-AKI. Moreover, abnormal physiological indicators and comorbidities, such as high serum osmotic pressure and a Syntax score of II, are also related to CI-AKI [6, 7]. CI-AKI increases the average length of hospital stay, accelerates the occurrence of end-stage renal disease and seriously affects the clinical prognosis of PCI in patients with coronary heart disease, such as the occurrence of major adverse cardiovascular events (MACEs), major adverse cerebrovascular events (MACCEs) and cardiac death (CD) [8].

Albumin is the major protein of human plasma. Plasma albumin has many physiological properties, such as antioxidant, anti-inflammatory, anticoagulant, and anti-platelet aggregation activity. Low serum albumin concentrations may be caused by liver damage during acute inflammation or increased renal excretion, malnutrition, increased catabolism, intestinal loss, severe volume overload, and escape to interstitial spaces [9]. Low serum albumin levels are independently associated with the development of various diseases, such as e myocardial infarction (MI), coronary artery disease (CAD), stroke, CI-AKI, hip fracture, and malignancy [10–13]. In patients with acute coronary syndrome, the serum albumin levels are significantly lower in those with CI-AKI than in those without CI-AKI, and the serum albumin level is an independent predictor of CI-AKI [12]. Preprocedural levels of prealbumin were independently associated with an increased risk of CI-AKI and long-term mortality in elderly patients undergoing elective PCI [14]. As indicators of nutritional status and inflammatory factors, perioperative albumin levels and postoperative effects were explored. Several previous studies have shown that perioperative albumin levels are significantly associated with postoperative complications, such as myocardial infarction, in patients with malignancies, including colorectal cancer, gastric cancer, and lung cancer, and in noncancer patients [15–19]. Similarly, the relationship between perioperative albumin levels and adverse outcomes after abdominal surgery has been discussed [20, 21].

However, there are few studies on the relationship between perioperative serum albumin (delta albumin or &Alb) and the occurrence of CI-AKI in patients with coronary heart disease after PCI. The present study was designed to examine the correlation between &Alb and CI-AKI in patients who underwent PCI.

## Methods

### Study population

**Fig. 1:**
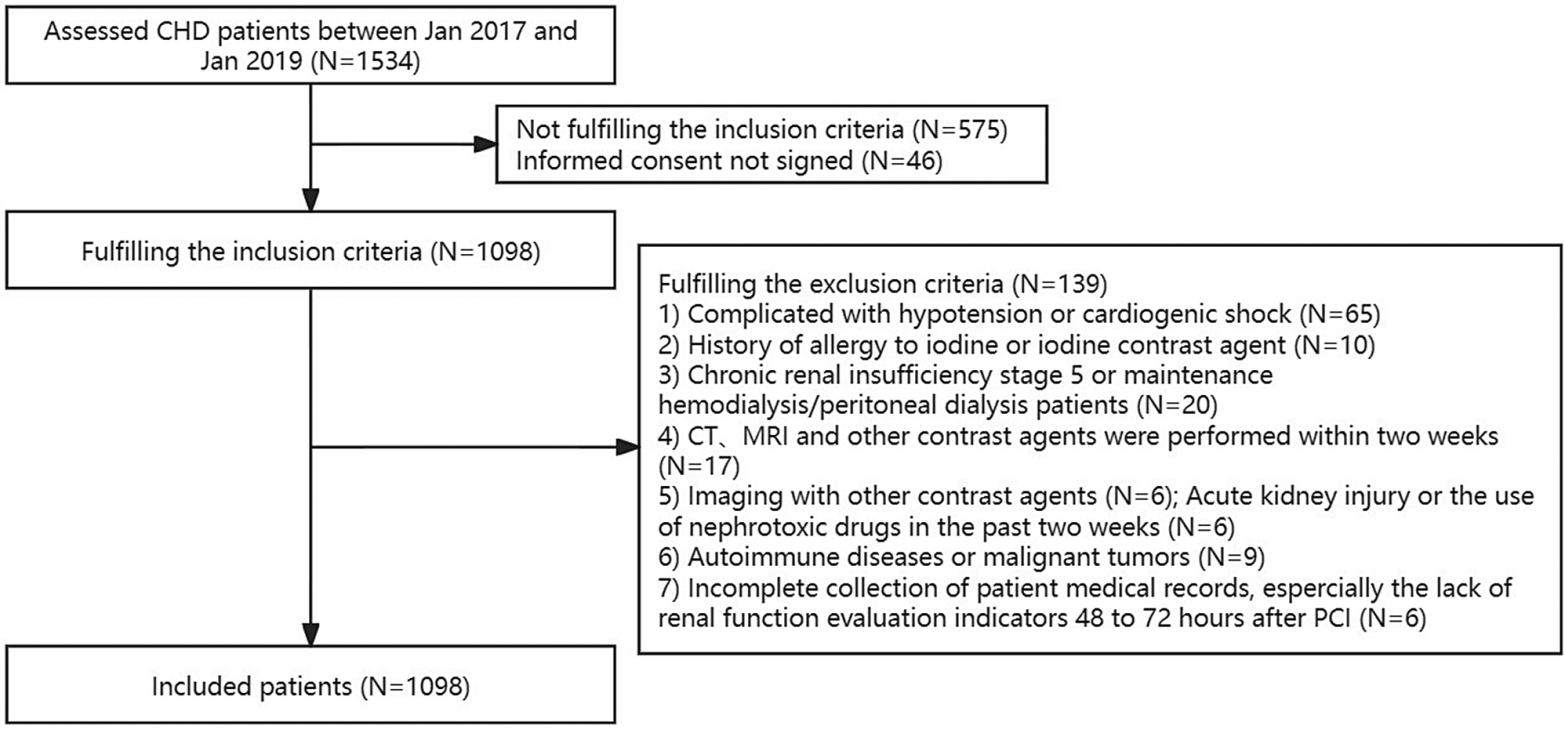
Flow diagram of patient selection

Patients who received their first PCI treatment between January 2017 and January 2019 were selected consecutively as potential participants in the Department of Cardiology, Zhongda Hospital, School of Medicine, Southeast University (see Fig. 1). The inclusion criteria were as follows: adult patients aged ≥ 18 years and patients who agreed to participate in the present study. The exclusion criteria were as follows: (1) complicated hypotension or cardiogenic shock; (2) history of allergy to iodine or iodine contrast agent; (3) stage 5 chronic renal insufficiency or maintenance haemodialysis/peritoneal dialysis; (4) CT scan, MRI scan, angiography or other contrast agent application within two weeks before inclusion; imaging examination using other contrast agents expected to be performed during the study period (except for the elective PCI 1 week later, the same contrast agent was required); (5) acute kidney injury or the use of nephrotoxic drugs in the past two weeks; (6) inflammatory diseases, autoimmune diseases, liver insufficiency, thyroid dysfunction, malignant tumours, or infectious diseases; and (7) incomplete collection of patient medical records, especially those lacking renal function evaluation indicators 48 to 72 h after PCI.

### Data collection

Each patient’s medical history, symptoms, signs, examination, and test results were recorded in detail. The data included age, sex, smoking status, hypertension status, diabetes status, atrial fibrillation status, stroke status, and body mass index at admission. The left ventricular ejection fraction (LVEF), routine blood examination, biochemistry, basic cardiovascular medication information, and volume of contrast media were recorded. Postoperative ALB was detected 48 to 72 h after PCI. Based on the coronary angiography results, the severity of coronary artery disease was evaluated by the Gensini score [22], and multivessel disease was defined as ≥ 50% diameter stenosis in at least 2 major coronary arteries.

### Diagnostic criteria for coronary heart disease (CHD)

The diagnostic criteria for CHD depend on typical angina pectoris symptoms, ECG changes, and the determination of myocardial injury markers. Coronary angiography is the gold standard for diagnosis, and coronary CTA can also be used for preliminary screening of coronary heart disease. (1) Chest pain symptoms: most of these symptoms involve crushing pain located in the retrosternal and precordial areas, which are related to activity and emotional agitation. The duration of nitrate drug release varies among the different types of drugs, and the degree of nitrate drug relief varies. (2) ECG manifestations: the ECG manifestations of UA and NSTEMI patients are transient ST segment (elevated or lowered) and T wave (flat or inverted) changes during the onset of chest pain. The ECG of STEMI patients can show ST-segment elevation, wide and deep Q waves, T-wave inversion, and so on. (3) Myocardial injury markers: for patients with STEMI and NSTEMI, myocardial injury markers are elevated at the corresponding time. However, the levels of markers of myocardial injury in UA patients were normal or slightly elevated. (4): Coronary angiography: coronary angiography is the gold standard for diagnosis. Coronary CTA can also be used for preliminary screening of CHD.

### Diagnostic criteria for CI-AKI

CI-AKI was defined as a greater than 25% or 44.2 µmol/L (0.5 mg/dL) increase in serum creatinine from baseline during the first 48 to 72 h after contrast exposure [4, 5].

### Clinical medication and CI-AKI prevention strategy

All patients received loading doses of double antiplatelet aggregation drugs (aspirin 300 mg and clopidogrel 300 mg or ticagrelor 180 mg) before surgery, and dual antiplatelet therapy (aspirin 100 mg·d^− 1^ and clopidogrel 75 mg·d^− 1^ or ticagrelor 180 mg·d^− 1^) continued for at least 12 months. The use of other medications (e.g., 𝛽-receptor blockers, angiotensin-converting enzyme inhibitors or angiotensin receptor blockers, nitrate esters, calcium antagonists, and statins) was left to the discretion of individual cardiologists. Adequate hydration remains the main means of preventing CI-AKI; at present, the best hydration solution is still inconclusive, and sodium chloride solution (NaCl) is widely used. Because the study population included several patients with cardiac insufficiency, the clinician was responsible for the decision regarding whether to hydrate according to the specific situation of each patient. In the case of hydration, the following requirements should be met: 3–12 h before surgery and 6–24 h after surgery. Isotonic crystal solution should be given intravenously at a rate of 1.0 mL·kg^−1^·h^−1^-1.5 mL·kg^−1·^h^−1^. The use of nephrotoxic drugs during the study (including large-dose loop diuretics, nonsteroidal anti-inflammatory drugs other than aspirin, aminoglycosides, amphotericin B, and traditional Chinese medicine containing aristolochic acid) should be avoided.

### Follow-up

The primary endpoint was the occurrence of major adverse cardiovascular events (MACEs), defined as the composite of all-cause death, nonfatal myocardial infarction, and target vessel revascularization (PCI or bypass surgery).

### Statistical analysis

A power analysis revealed that 782 patients needed to participate to achieve 80% power for detecting a difference between the two groups. To account for anticipated dropouts with no postbaseline data, 862 patients were required to begin the study, necessitating at least 431 patients for each group. Statistical analysis of the data was performed using SPSS 27.0 software. Normally distributed data are expressed as the mean (x) ± standard deviation (s), and comparisons between two groups were performed by an independent sample t test. The data with skewed distribution are expressed as M (Q1, Q3), and the Mann‒Whitney U test was used for comparisons between two groups. Enumeration data are expressed as a rate or constituent ratio, and comparisons between two groups were performed by the chi-square test or Fisher’s exact probability method. Multivariate logistic regression analysis was employed to analyse the correlation between &Alb and CI-AKI incidence. The receiver operating characteristic (ROC) curve was used to determine the optimal cut-off value and area under the curve (AUC) of &Alb for predicting CI-AKI after PCI. Survival analysis was performed using Kaplan‒Meier survival analysis. The log-rank test was used for comparisons between two groups. Multivariate Cox regression was used to analyse the predictors of MACEs in CHD patients during the 1-year follow-up after PCI. *P* < 0.05 was considered to indicate statistical significance according to a two-tailed test.

## Results

### Baseline characteristics

A total of 959 patients who underwent PCI were included in this study. The average age was 67 ± 10.5 years [range: 60–75]. Among these patients, 603 (62.9%) were male, 703 (73.3%) had hypertension, and 316 (33.0%) had diabetes, among whom 147 had experienced CI-AKI. The incidence of CI-AKI in patients who underwent PCI was 15.3% (147/959).

Patients were divided into 2 groups based on the optimal cut-off value of &Alb: a high &Alb group (&Alb ≥ 4.55 g/L, a total of 440 patients) and a control group (&Alb < 4.55 g/L, a total of 519 patients). The incidence of CI-AKI in the high &Alb group was significantly greater than that in the low group [23.6% (104/440) versus 8.3% (43/519), *P* < 0.01]. A comparison of the clinical data between the high &Alb group and the low group is shown in Table 1. The high &Alb group included more males, older individuals, and greater proportions of smokers and individuals with hypertension (*P* < 0.05).

**Table 1:** Baseline characteristics of the two groups

Variables	High & Alb group(*n* = 440)	Low & Alb group(*n* = 519)	*P* value
Male, n (%)	293 (66.6%)	310 (59.7%)	0.029
Age(years)	68 ± 10	66 ± 11	0.023
BMI (kg·m^2 − 1^)	24.89 ± 3.2	25 ± 3.4	0.549
Systolic BP (mmHg)	135.50 ± 18.95	137.97 ± 21.03	0.053
Diastolic BP (mmHg)	76.75 ± 12.74	76.35 ± 12.98	0.713
Smoking, n (%)	136 (30.9%)	113 (21.8%)	<0.01
Hypertension, n (%)	308 (70.0%)	395 (76.1%)	0.033
Diabetes, n (%)	145 (33.0%)	171 (32.9%)	0.998
Atrial fibrillation, n (%)	46 (10.5%)	36 (6.9%)	0.052
LVEF (%)	46 (10.5%)	36 (6.9%)	0.375
Stroke, n (%)	93 (21.1%)	123 (23.7%)	0.344
Medication, n (%)			
Aspirin	433 (98.4%)	506 (97.5%)	0.324
ACEI/ARB	240 (54.5%)	295 (56.8%)	0.476
β-blocker	347 (78.9%)	403 (77.6%)	0.650
Statins	401 (99.8%)	381 (99.7%)	0.662
Angiography			
Gensini score	64.7 ± 37.52	66.2 ± 38.36	0.388
Contrast dose (mL)	105.6 ± 22.31	109.3 ± 20.26	0.664
Vessels, *n*	2.16 ± 0.808	2.20 ± 0.789	0.281
Multi-vessel, *n* (%)	184 (41.8%)	225 (43.4%)	0.284
Stents, *n*	1.59 ± 0.708	1.50 ± 0.666	0.103
Multi-stent, *n* (%)	159 (36.1%)	173 (33.3%)	0.364
hydration, *n* (%)	104 (68.5)	338 (65.1)	0.386
CI-AKI, *n* (%)	104 (23.6%)	43 (8.3%)	<0.01

*Note* Data are presented as the mean ± Standard deviation, mean (25% quartile-75% quartile) or n (%)

*Abbreviations* BMI, body mass index; BP, blood pressure; LVEF, left ventricular ejection fraction; ACEI, angiotensin-converting enzyme inhibitor; ARB, angiotensin receptor blocker; CI-AKI, contrast-induced acute kidney injury; &Albumin, Perioperative Serum Albumin

A comparison of the laboratory parameters between the 2 groups is shown in Table 2. Patients in the high &Alb group had significantly greater baseline platelet counts, indirect bilirubin levels, and uric acid levels than did those in the low group (*P* < 0.05). However, patients in the high &Alb group had significantly lower total cholesterol, low-density lipoprotein cholesterol (LDL-C) and HDL-C levels than patients in the low &Alb group (*P* < 0.05).

**Table 2:** Baseline laboratory characteristics of the two groups

Variables	High & Alb group(*n* = 440)	Low & Alb group(*n* = 519)	*P* value
RBC (×10^12^·L^− 1^)	4.48 ± 0.55	4.45 ± 0.58	0.672
WBC (×10^9^·^·^L^− 1^)	7.05 ± 2.42	6.98 ± 2.23	0.470
HGB (g·L^− 1^)	137.38 ± 16.30	135.35 ± 17.90	0.068
PLT (×10^9^·L^− 1^)	213.77 ± 67.30	195.8 ± 58.87	<0.01
NEU (×10^9^·L^− 1^)	4.63 ± 1.61	4.71 ± 1.92	0.435
LYM (×10^9^·L^− 1^)	1.71 ± 0.75	1.67 ± 0.61	0.357
MON (×10^9^·L^− 1^)	0.42 ± 0.246	0.41 ± 0.178	0.203
DBIL (µmol·L^− 1^)	3.27 ± 2.14	3.89 ± 2.99	0.318
IBIL (µmol·L^− 1^)	9.29 ± 4.46	8.61 ± 4.29	0.016
ALT (U·L^− 1^)	26.15 ± 23.62	26.17 ± 20.66	0.988
AST (U·L^− 1^)	26.95 ± 25.62	27.46 ± 21.33	0.740
Glucose (mmol·L^− 1^)	6.64 ± 2.52	7.10 ± 2.98	0.011
SCr (µmol·L^− 1^)	84.55 ± 34.49	81.39 ± 23.39	0.102
Uric acid (µmol·L^− 1^)	375.01 ± 112.07	358.70 ± 100.67	0.019
Triglyceride (mmol·L^− 1^)	1.67 ± 0.97	1.84 ± 1.61	0.023
TC (mmol·L^− 1^)	4.28 ± 1.07	4.62 ± 1.24	<0.01
HDL-C (mmol·L^− 1^)	1.09 ± 0.24	1.15 ± 0.34	0.004
LDL-C (mmol·L^− 1^)	2.60 ± 0.89	2.84 ± 0.99	<0.01
Preoperative albumin (g·L^− 1^)	39.53 ± 3.96	39.43 ± 4.31	0.705
Postoperative albumin (g·L^− 1^)	31.82 ± 4.53	38.77 ± 5.47	<0.01

*Note* Data are the mean ± Standard deviation, mean (25% quartile-75% quartile) or n (%)

*Abbreviations* RBC, red blood cells; WBC, white blood cells; HGB, hemoglobin; PLT, platelet count; NEU, neutrophil count; LYM, lymphocyte count; MON, monocyte count; DBIL, direct bilirubin; IBIL, indirect bilirubin; ALT, alanine aminotransferase; AST, alanine aminotransferase; SCr, Serum creatinine; TC, Total cholesterol; HDL-C, high-density lipoprotein cholesterol; LDL-C, low-density lipoprotein cholesterol; & Albumin, Perioperative Serum Albumin

### Correlations between &Alb and CI-AKI

**Fig. 2:**
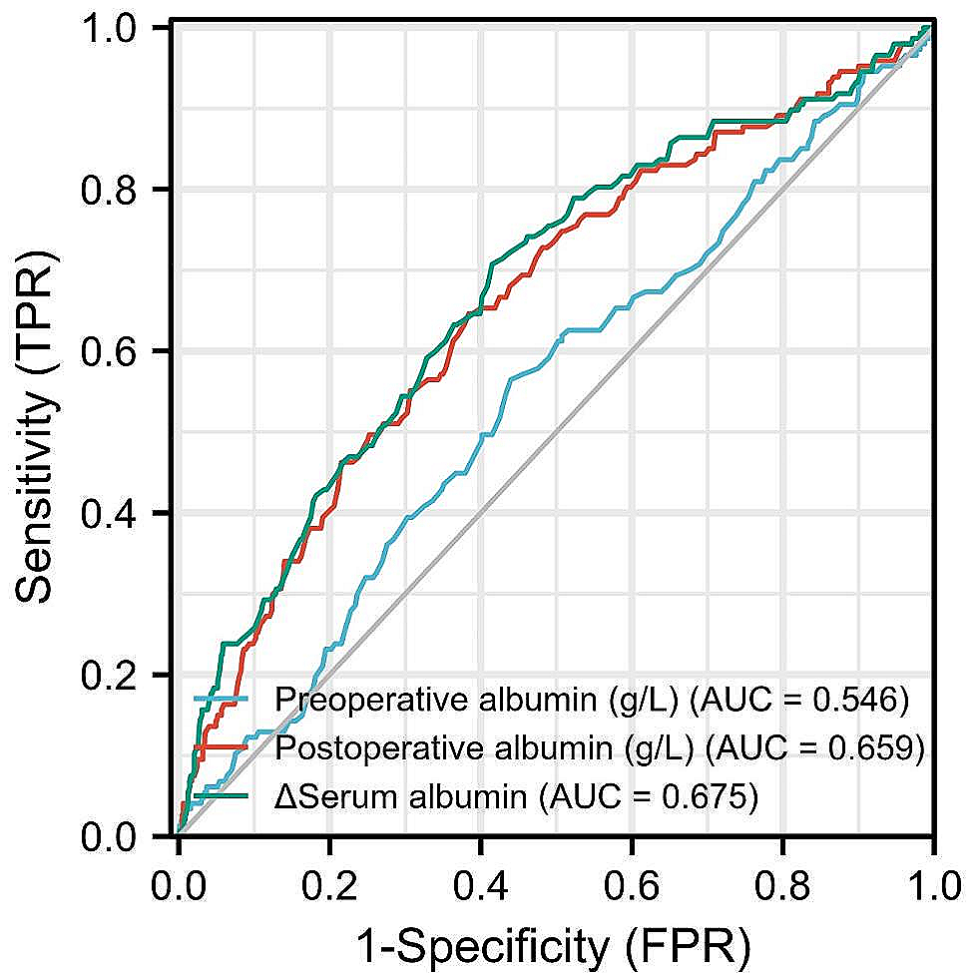
ROC curve of various serum albumin predicting CI-AKI

Pearson correlation analysis revealed that &Alb levels were linearly correlated with CI-AKI (*r* = 0.212, *P* < 0.001). Moreover, ROC curve analysis and AUC values (Fig. 2; Table 3) revealed that the AUC of &Alb for CI-AKI after PCI was 0.675 [95% confidence interval (CI): 0.627–0.724, *P* < 0.001]. The best cut-off value was 4.55 g/L, the sensitivity was 70.7%, and the specificity was 58.5%. The postoperative serum ALB concentration for CI-AKI patients after PCI was 0.659 [95% CI: 0.626–0.723, *P* < 0.001], the best cut-off value was 4.55 g/L, the sensitivity was 70.7%, and the specificity was 58.5%.

**Table 3:** Area under curve between valueariables

Variables	AUC	Cut-off value	Sensitivity	Specificity	*P* value	95% CI
& Alb	0.675	4.55	0.707	0.585	<0.01	0.626–0.723
Preoperative albumin (g·L^− 1^)	0.546	39.15	0.565	0.56	0.075	0.495–0.597
Postoperative albumin g·L^− 1^)	0.659	34.25	0.646	0.616	<0.01	0.626–0.723

*Note* AUC = area under curve; 95%CI = 95% confidence interval; CI-AKI, contrast-induced acute kidney injury. & Albumin, Perioperative Serum Albumin

Multivariate logistic regression analysis revealed that high &Alb [odds ratio (OR) 2.495, 95% CI: 1.277–4.874, *P* = 0.007], male sex (OR 0.616, 95% CI: 0.389–0.976, *P* = 0.039), serum creatinine level (OR 1.021, 95% CI: 1.015–1.027, *P* < 0.001), triglyceride level (OR 0.775, 95% CI: 0.627–0.958, *P* = 0.019), postoperative albumin level (OR 0.911, 95% CI: 0.883–0.940, *P* < 0.001), hypoproteinaemia after surgery (OR 2.497, 95% CI: 1.361–4.582, *P* = 0.003), and triglyceride level (OR 0.775, 95% CI: 0.627–0.958, *P* = 0.019) were independent risk factors for the development of CI-AKI.

DeLong’s test showed that the diagnostic efficiency of the preoperative albumin level was significantly worse than that of the postoperative albumin level (*P* < 0.01). The diagnostic efficiency of the preoperative albumin level was lower than that of the preoperative &Alb level, and the difference was statistically significant (*P* < 0.01). The diagnostic efficiency of postoperative ALB was worse than that of &Alb, but the difference was not statistically significant (*P* = 0.417).

### Analysis of &Alb and long-term clinical outcomes

**Fig. 3:**
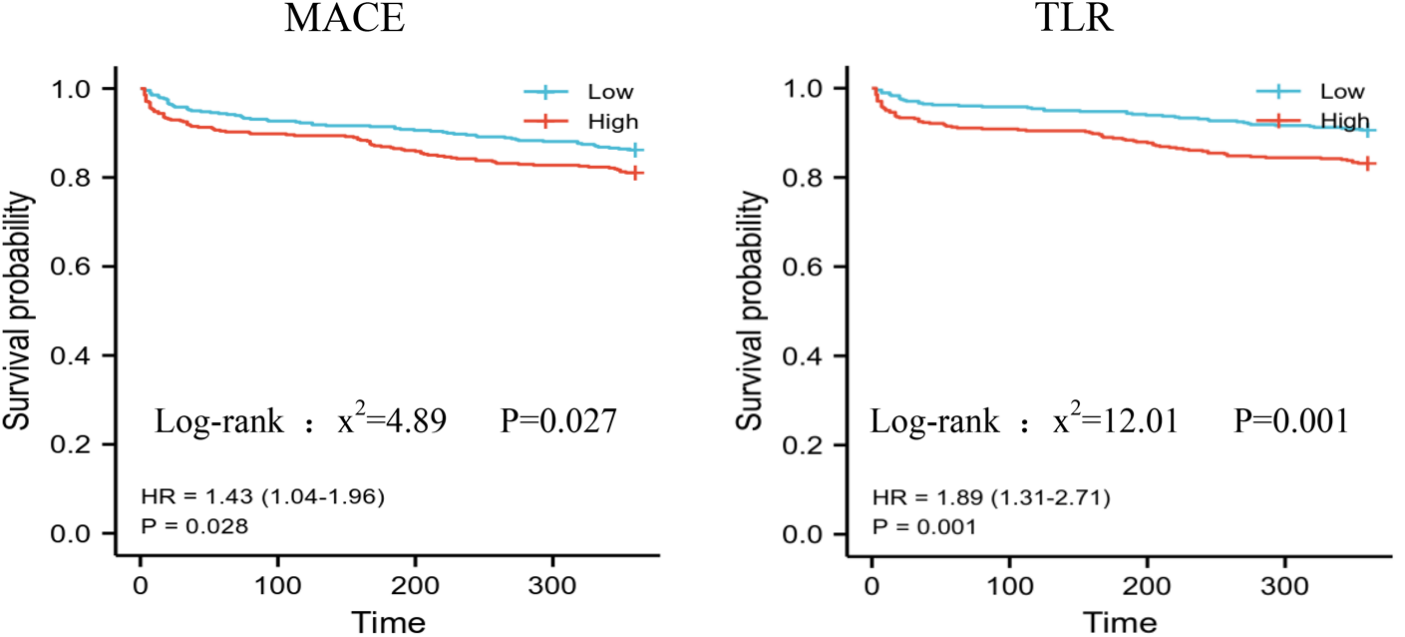
The Kaplan-Meier survival analysis curve of clinical outcomes after 1-year follow-up among the 2 groups. *Note* Blue line, low &Albumin group; red line, high &Albumin group

All patients completed a 1-year clinical follow-up. Patients with high &Alb levels (≥ 4.55 g/L) had worse clinical outcomes, with a greater incidence of primary endpoints [18.6% (82/440) vs. 14.5% (75/519), *P* = 0.022]. No significant differences were found in all-cause death (1.3% vs. 0.1%, *P* = 0.087) or nonfatal myocardial infarction (4.6% vs. 3.4%, *P* = 0.342). However, the incidence of target vascular revascularization (16.6% vs. 10.2%, *P* = 0.004) was significantly greater in the high &Alb group. Kaplan-Meier curves are shown in Fig. 3.

### Evaluation of the prognostic performance of serum &Alb and CI-AKI for MACEs

Cox regression analysis was performed to evaluate the independent predictors of MACEs in patients at the 1-year follow-up, which showed that CI-AKI was an independent predictor of primary endpoint outcomes at the 1-year follow-up [hazard ratio (HR) 1.521, 95% CI: 1.036–2.233, *P* = 0.032]. Another variable showing an independent prognostic impact was multivessel disease (HR 3.145, 95% CI: 1.839–5.377, *P* < 0.01). The results of the Cox regression analysis are presented in Fig. 3.

## Discussion

In this study, we investigated the predictive value of perioperative serum albumin levels for the occurrence of CI-AKI in CHD patients after PCI. We found that the incidence of CI-AKI in the high &Alb group was significantly greater than that in the low &Alb group. The AUC of &Alb for detecting CI-AKI after PCI was 0.675 (95% CI = 0.627–0.724, *P* < 0.001), with a sensitivity of 70.7%. We demonstrated that decreased perioperative albumin levels (&Alb ≥ 4.55 g · L^− 1^) were an independent risk factor for CI-AKI and an important predictor of MACEs 1 year after PCI in patients with CHD; &Alb ≥ 4.55 g · L^− 1^ could not only be used for the early identification of CI-AKI in the short term after PCI in patients but also had high predictive value for MACEs 1 year after PCI.

Contrast agents are commonly used to enhance imaging, particularly in computed tomography and magnetic resonance imaging, as well as in coronary angiography (CAG) and percutaneous coronary intervention (PCI) [12, 23]. In patients undergoing PCI, CI-AKI has emerged as a serious complication closely related to clinical adverse events, increasing morbidity and mortality rates [3, 5, 24]. The clinical and surgical prognosis of patients with CHD is significantly improved by the use of coronary stenting [25]. However, the incidence of CI-AKI is increasing annually, and CI-AKI has become a critical complication of coronary revascularization. Our results showed that CI-AKI increases the occurrence of MACE and significantly affects the clinical prognosis of patients after PCI. The pathogenesis of CI-AKI is complex, and the specific pathophysiologic basis remains unclear. At present, this effect is primarily attributed to intrarenal vasoconstriction, the production of reactive oxygen species, and direct tubular injury. After intravascular injection of contrast media, various factors can lead to renal vasoconstriction, including antidiuretic hormone (ADH), adenosine, and endothelin-1 (ET-1) [26]. These factors transiently increase renal arterial blood flow, followed by persistent severe contraction, eventually causing renal hypoperfusion, renal medullary ischaemia, and hypoxia [27]. Oxidative stress injury may also be one of the pathophysiological mechanisms of CI-AKI. The contrast agent can lead to a reduction in blood supply to the renal medulla, causing an imbalance between metabolic demand and blood supply in the thick ascending limb of the Henle loop in the outer medullary layer. This imbalance results in the generation of superoxide, leading to oxidative necrosis of the renal tubules [28, 29]. Direct cytotoxicity of contrast agents in vascular endothelial cells leads to elevated levels of endothelin and adenosine, reduced nitric oxide and prostaglandins [30], and increased fluid viscosity in vascular and tubular cells. Although the majority of contrast media-induced kidney damage can return to normal levels within 1 to 4 weeks, several risk factors, such as CKD and hypotension, may result in the loss of functional nephrons and impairment of renal function. As the dose of contrast medium increases, the regenerative capacity of tubular epithelial cells is compromised, leading to potential fibrosis and permanent loss of function in some renal units [31, 32]. Contrast agents act as allergens, triggering systemic allergic reactions and renal immune inflammatory responses and playing a role in the mechanisms of CI-AKI [28]. Systemic inflammation can render the kidney more vulnerable to local inflammation induced by iodinated contrast agents after angiography, thereby exacerbating the development of contrast-induced acute kidney injury.

Vascular remodelling induced by oxidative stress and the secondary inflammatory response is the central link in the chain of events in CHD. Endothelial dysfunction and an imbalance in smooth muscle phenotype conversion are two key factors in vascular remodelling. Atherosclerosis is associated with several susceptibility factors for CI-AKI and results in the release of reactive metabolites that can induce haemodynamic and inflammatory changes, further compromising renal blood flow. As one of the routine examinations for hospitalized patients, serum albumin plays a crucial role in binding and transporting endogenous and exogenous substances in the body. It helps maintain stable colloid osmotic pressure in the blood, eliminates free radicals harmful to the body, and inhibits platelet aggregation to achieve an anticoagulant effect [33]. Low levels of serum albumin can lead to the cytotoxicity and strong atherogenic effects of oxidatively deformed LDL [34]. Conversely, reduced albumin expression can increase blood viscosity, thereby promoting the progression of CHD and disrupting normal vascular endothelial function, ultimately impairing cardiac function. As indicators of nutritional status and inflammatory factors, perioperative albumin levels and postoperative outcomes were investigated. Several previous studies have shown that perioperative albumin levels are significantly associated with postoperative complications in patients with malignancies, such as colorectal cancer, gastric cancer, and lung cancer, as well as in noncancer patients [13, 15, 17, 18]. Similarly, the relationship between perioperative albumin levels and adverse outcomes after abdominal surgery has been discussed [17, 18].

The incidence of CI-AKI in patients after PCI was found to be as high as 15.3% in this study, making early identification of patients at risk for CI-AKI extremely important. In terms of CI-AKI, our findings are largely consistent with the results from a clinical study in which 890 ACS patients were enrolled and underwent primary PCI, indicating that lower serum albumin levels may predict CI-AKI development after primary PCI in ACS patients [8]. Moreover, multivariate logistic regression analysis revealed that a high &Alb level (OR 2.495, 95% CI: 1.277–4.874, *P* = 0.007) can predict CI-AKI development after primary PCI in CHD patients. Receiver operating characteristic curve analysis revealed that the &Alb level is an accurate predictor for the development of CI-AKI. The area under the curve was 0.675 for the baseline &Alb level (95% CI: 0.627–0.724, *P* < 0.001). The optimal cut-off point of &Alb was 4.55 g/L, with a sensitivity of 70.7% and a specificity of 58.5%. These findings suggest that a reduction in perioperative serum albumin levels can serve as a predictor of CI-AKI following PCI. This can aid in the early identification of patients undergoing PCI who are at high risk of developing CI-AKI, laying the groundwork for implementing preventive strategies. For patients with decreased serum albumin levels during the perioperative period, it is necessary to maintain sufficient mean arterial pressure, supplement albumin, and administer medication to reduce extravascular protein leakage. More samples and studies are needed to further prove this point.

By comparing the baseline data of the two groups of patients, we also found that the patients in the high &Alb group were older and had lower body mass index values. This observation suggests that the postoperative reduction in albumin levels was more pronounced in older patients than in younger patients, which may be associated with low food intake and poor nutritional status in elderly patients. Serum albumin levels are primarily used to assess malnutrition and chronic diseases. A low level of serum albumin can result in decreased plasma colloid osmotic pressure, excessive fluid accumulation in the interstitial space, reduced effective circulating blood volume, microcirculatory disturbances, hypoperfusion of vital organs, and potentially multiple organ dysfunction. This suggests that we should direct more attention to the nutritional status of elderly patients before surgery.

At the 1-year follow-up, the incidence of MACEs was greater in the high &Alb group than in the low &Alb group. Cox regression analysis revealed that CI-AKI was an independent predictor of the primary endpoint outcome, indicating that CI-AKI is associated with an increase in MACEs in CHD patients. CI-AKI is strongly linked to a poor prognosis. The results of this study highlight the importance of CI-AKI in the incidence of MACEs.

This study has several limitations. First, all patients in the current study came from one hospital, so the sample size was small, and selection bias was inevitable. This issue needs to be addressed by collecting data from a larger sample size to further demonstrate its impact. Furthermore, many factors can affect albumin levels and/or the incidence of myocardial infarction. Although many disease states that may affect the final results were excluded from this study and multifactor adjustments were performed to reduce bias, there may still be other confounding factors that were not adjusted for during enrolment, which may have affected the results.

## Conclusion

Serum albumin levels measured before and after surgery can help identify CI-AKI after PCI and predict long-term prognosis. According to the extent of perioperative albumin reduction, clinicians are encouraged to implement additional CI-AKI prevention measures for CI-AKI patients and closely monitor high-risk patients to improve patient prognosis. This approach holds significant clinical relevance and practical value.

## Data Availability

Data related to this manuscript can be made available from the corresponding author upon reasonable request.

## References

[1] Kooiman J, van de Peppel WR, Sijpkens YW (2015). No increase in kidney Injury Molecule-1 and Neutrophil Gelatinase-Associated Lipocalin excretion following intravenous contrast enhanced-CT. Eur Radiol.

[2] Ali ZA, Karimi Galougahi K, Nazif T (2016). Imaging- and physiology-guided percutaneous coronary intervention without contrast administration in advanced renal failure: a feasibility, safety, and outcome study. Eur Heart J.

[3] Rudnick MR, Leonberg-Yoo AK, Litt HI, Cohen RM, Hilton S, Reese PP (2020). The controversy of contrast-Induced Nephropathy with intravenous contrast: what is the risk. Am J Kidney Dis.

[4] Stacul F, van der Molen AJ, Reimer P (2011). Contrast induced nephropathy: updated ESUR Contrast Media Safety Committee guidelines. Eur Radiol.

[5] van der AJ, Reimer P, Dekkers IA (2018). Post-contrast acute kidney injury - part 1: definition, clinical features, incidence, role of contrast medium and risk factors: recommendations for updated ESUR Contrast Medium Safety Committee guidelines. Eur Radiol.

[6] Rencuzogullari I, Çağdaş M, Karakoyun S (2018). Association of Syntax score II with contrast-induced Nephropathy and Hemodialysis requirement in patients with ST Segment Elevation myocardial infarction undergoing primary percutaneous coronary intervention. Korean Circ J.

[7] Yildiz I, Yildiz PO, Rencuzogullari I (2019). Association of Serum Osmolarity with Contrast-Induced Nephropathy in patients with ST-Segment Elevation myocardial infarction. Angiology.

[8] Levitt DG, Levitt MD (2016). Human serum albumin homeostasis: a new look at the roles of synthesis, catabolism, renal and gastrointestinal excretion, and the clinical value of serum albumin measurements. Int J Gen Med.

[9] Djoussé L, Rothman KJ, Cupples LA, Levy D, Ellison RC (2002). Serum albumin and risk of myocardial infarction and all-cause mortality in the Framingham offspring study. Circulation.

[10] Gillum RF, Ingram DD, Makuc DM (1994). Relation between serum albumin concentration and stroke incidence and death: the NHANES I epidemiologic follow-up study. Am J Epidemiol.

[11] Wang M, Liu J, Ma C (2012). Synergistic association of serum albumin and globulin with coronary heart disease. J Atheroscler Thromb.

[12] Murat SN, Kurtul A, Yarlioglues M (2015). Impact of serum albumin levels on contrast-Induced Acute kidney Injury in patients with Acute coronary syndromes treated with percutaneous coronary intervention. Angiology.

[13] Hardt J, Pilz L, Magdeburg J, Kienle P, Post S, Magdeburg R (2017). Preoperative hypoalbuminemia is an independent risk factor for increased high-grade morbidity after elective rectal cancer resection. Int J Colorectal Dis.

[14] You ZB, Lin KY, Zheng WP (2018). Association of prealbumin levels with contrast-induced acute kidney injury in elderly patients with elective percutaneous coronary intervention. Clin Interv Aging.

[15] Uppal U, Al-Niaimi A, Rice LW (2013). Preoperative hypoalbuminemia is an independent predictor of poor perioperative outcomes in women undergoing open surgery for gynecologic malignancies. Gynecol Oncol.

[16] Bohl DD, Shen MR, Hannon CP, Fillingham YA, Darrith B, Della Valle CJ (2017). Serum albumin predicts survival and postoperative course following surgery for geriatric hip fracture. J Bone Joint Surg Am.

[17] Labgaa I, Joliat GR, Kefleyesus A (2017). Is postoperative decrease of serum albumin an early predictor of complications after major abdominal surgery? A prospective cohort study in a European centre. BMJ Open.

[18] Li P, Li J, Lai Y (2018). Perioperative changes of serum albumin are a predictor of postoperative pulmonary complications in lung cancer patients: a retrospective cohort study. J Thorac Dis.

[19] Karakayali M, Omar T, Artac I (2023). The prognostic value of HALP score in predicting in-hospital mortality in patients with ST-elevation myocardial infarction undergoing primary percutaneous coronary intervention. Coron Artery Dis.

[20] Gensini GG (1983). A more meaningful scoring system for determining the severity of coronary heart disease. Am J Cardiol.

[21] Issangya CE, Msuya D, Chilonga K (2020). Perioperative serum albumin as a predictor of adverse outcomes in abdominal surgery: prospective cohort hospital based study in Northern Tanzania. BMC Surg.

[22] Lee SE, Jang JE, Kim HS (2019). Mesenchymal stem cells prevent the progression of diabetic nephropathy by improving mitochondrial function in tubular epithelial cells. Exp Mol Med.

[23] Kis M, Guzel T (2022). Relationship between Hemoglobin A1c and Fractional Flow Reserve Lesion Severity in non-diabetic patients. J Coll Physicians Surg Pak.

[24] Güzel T, Aktan A, Demir M, Özbek M, Aslan B. Relationship between contrast-induced nephropathy and long-term mortality after percutaneous coronary intervention in patients with chronic coronary total occlusion. Rev Assoc Med Bras (1992). 2022. 68(8): 1078–1083. 10.1590/1806-9282.20220283 PMC9574976 36000604

[25] Guzel T, Bilik MZ, Arslan B, Kilic R, Aktan A (2021). The effect of atherogenic plasma index on collateral development in patients with chronic coronary total occlusion. Experimental Biomedical Res.

[26] Chang CF, Lin CC (2013). Current concepts of contrast-induced nephropathy: a brief review. J Chin Med Assoc.

[27] Hossain MA, Costanzo E, Cosentino J (2018). Contrast-induced nephropathy: pathophysiology, risk factors, and prevention. Saudi J Kidney Dis Transpl.

[28] Dugbartey GJ, Redington AN (2018). Prevention of contrast-induced nephropathy by limb ischemic preconditioning: underlying mechanisms and clinical effects. Am J Physiol Ren Physiol.

[29] Liu LY, Liu Y, Wu MY, Sun YY, Ma FZ (2018). Efficacy of atorvastatin on the prevention of contrast-induced acute kidney injury: a meta-analysis. Drug Des Devel Ther.

[30] Pisani A, Riccio E, Andreucci M et al. Role of reactive oxygen species in pathogenesis of radiocontrast-induced nephropathy. Biomed Res Int. 2013. 2013: 868321. 10.1155/2013/868321 PMC3891610 24459673

[31] Abizaid AS, Clark CE, Mintz GS (1999). Effects of dopamine and aminophylline on contrast-induced acute renal failure after coronary angioplasty in patients with preexisting renal insufficiency. Am J Cardiol.

[32] Sun G, Chen JY, Liu Y (2019). Contrast-Induced Nephropathy: further investigations about risk factors are required. Angiology.

[33] Tsirpanlis G, Bagos P, Ioannou D (2004). Exploring inflammation in hemodialysis patients: persistent and superimposed inflammation. A longitudinal study. Kidney Blood Press Res.

[34] Liu T, Lee SR (2021). Poor prognosis of contrast-Induced Nephropathy during Long Term follow up. Chonnam Med J.

